# Guillain-Barré Syndrome in a Patient With Cervical Myelopathy and Lumbar Spinal Stenosis Following a Fall: A Diagnostic Challenge

**DOI:** 10.7759/cureus.116588

**Published:** 2026-09-20

**Authors:** Mariam Ahmed, Muhammad Mohsin Khan, Ali Kamal Sulaiman, Tarek BinZabih

**Affiliations:** 1 Medicine, Qatar University, Doha, QAT; 2 Clinical Research, Dresden International University, Dresden, DEU; 3 Neurosurgery, Hamad Medical Corporation, Doha, QAT

**Keywords:** albuminocytologic dissociation, cervical myelopathy, diagnostic challenge, guillain-barré syndrome, lumbar spinal stenosis

## Abstract

Guillain-Barré syndrome (GBS) is an acute autoimmune disease characterized by symmetrical weakness that develops over a few days to four weeks. Its diagnosis can be challenging when symptoms overlap with other conditions, such as lumbar spinal stenosis. We report the case of a 55-year-old man who presented with sudden lower-extremity weakness and bladder incontinence following a fall after alcohol intake. Magnetic resonance imaging revealed cervical and lumbar spinal stenosis with compressive changes, which were initially considered the likely cause of his symptoms. However, the patient subsequently deteriorated, with investigations revealing positive results for *Klebsiella* and rhinovirus. Given the progression of his neurological deficits, GBS was suspected, and plasmapheresis was initiated. Cerebrospinal fluid (CSF) analysis demonstrated albuminocytologic dissociation, while nerve conduction studies (NCS) showed severe widespread sensorimotor axonal polyneuropathy, supporting the diagnosis. The patient showed significant neurological and functional improvement following treatment and rehabilitation. This case highlights the diagnostic challenges of differentiating GBS from cervical spondylotic myelopathy in patients with pre-existing spinal pathology. Precise history-taking, serial neurological examinations, and appropriate investigations, including CSF analysis and NCS, are essential to avoid misdiagnosis and facilitate early management and improved patient outcomes.

## Introduction

Guillain-Barré syndrome (GBS) is an acute immune-mediated peripheral neuropathy that commonly follows a respiratory or gastrointestinal infection [1]. It typically presents with progressive, symmetrical weakness and reduced or absent deep tendon reflexes. However, clinical presentation can vary, and early diagnosis may be challenging when other conditions can explain the neurological findings. Cervical spinal cord disease can also cause acute or progressive weakness, particularly in patients with pre-existing cervical stenosis who experience trauma [2]. Features such as hyperreflexia, spasticity, a sensory level, and bladder dysfunction may suggest a spinal cord lesion rather than GBS [3]. However, distinguishing the two can be difficult when significant degenerative changes are present on spinal imaging [4].

We report the case of a 55-year-old man with diabetes and spinal degenerative disease who developed bilateral lower-limb weakness and urinary incontinence after a fall. Cervical magnetic resonance imaging (MRI) showed significant C5-C6 spinal canal stenosis with cord compression and T2-weighted hyperintensity. Despite these findings, the patient subsequently developed progressive weakness, generalized areflexia, and respiratory deterioration. Cerebrospinal fluid (CSF) showed albuminocytologic dissociation, and nerve conduction studies (NCS) demonstrated severe motor and sensory axonal neuropathy. He was treated with plasma exchange for suspected GBS and subsequently showed neurological improvement. This case highlights the difficulty of distinguishing GBS from cervical spinal pathology and the importance of considering both conditions when imaging findings do not fully explain the clinical course.

## Case presentation

A 55-year-old man with long-standing, poorly controlled type 2 diabetes mellitus and a history of chronic mild back pain related to disc prolapse presented by ambulance following a sudden fall in the bathroom. The night before, he reported consuming a small amount of alcohol. According to the patient, the bilateral lower-limb weakness and urinary incontinence began after the fall. He initially denied sensory symptoms. On initial neurological assessment, he was alert and oriented with a Glasgow Coma Scale score of 15/15 and intact cranial nerves. Bilateral lower-limb power was markedly reduced, initially approximately 1/5. Early examination demonstrated reduced knee reflexes, which subsequently progressed to generalized areflexia. Sensation was initially preserved.

Subsequent examination demonstrated progression of weakness involving the upper limbs, particularly bilateral hand grip, while lower-limb weakness remained severe. At the time of neurological deterioration, power was approximately 1/5 at the hips, 1/5 at knee flexion with 3-/5 knee extension, 4-/5 ankle dorsiflexion and plantarflexion, and 4/5 extensor hallucis longus bilaterally. Bilateral hand grip was approximately 3-/5, with otherwise relatively preserved proximal upper-limb power. Reflexes were absent throughout the upper and lower limbs, while sensation remained intact.

MRI of the cervical spine showed significant C5-C6 spinal canal stenosis due to a broad-based disc protrusion, osteophyte formation, and ossification of the posterior longitudinal ligament, with associated spinal cord compression and intramedullary T2 hyperintensity at the level of maximal stenosis, consistent with compressive myelopathic change (Figure 1). Multilevel degenerative changes and neural foraminal stenosis were also present. Lumbar imaging demonstrated degenerative disc disease with significant canal stenosis, particularly at L3-L4 and L4-L5. These structural abnormalities initially provided a plausible explanation for the patient's acute neurological deficits, so he was admitted under neurosurgical care for further assessment.

**Figure 1:**
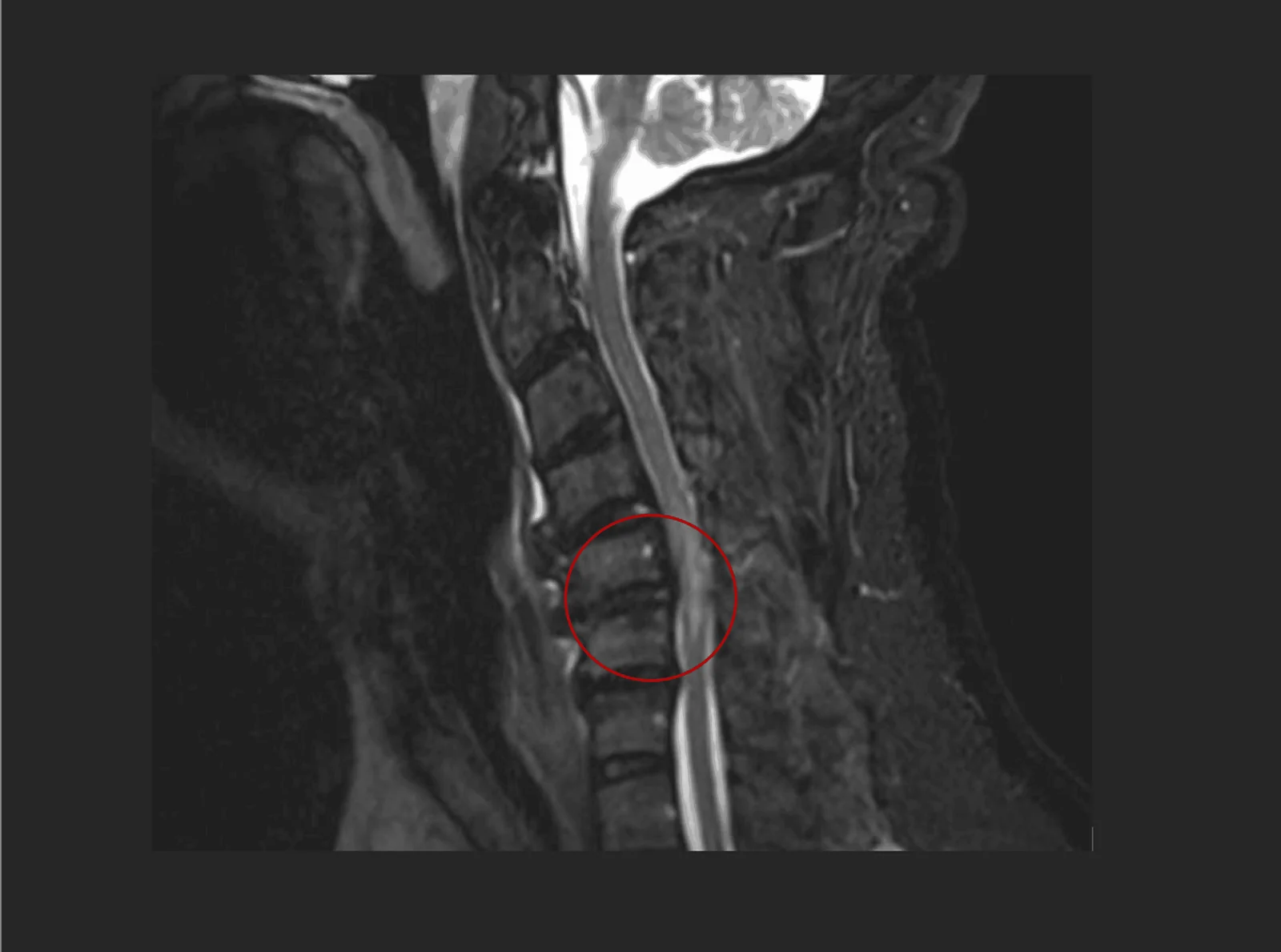
Sagittal T2-weighted MRI demonstrating cervical spinal canal stenosis at C5–C6 with associated spinal cord T2 signal abnormality. The red circle highlights the area of maximal stenosis and cord signal change.

During hospitalization, the patient subsequently developed progressive respiratory difficulty requiring non-invasive ventilatory support. At that time, he had severe bilateral lower-limb weakness, with areflexia of the lower limbs and preserved sensation. Respiratory deterioration occurred in the setting of concurrent infection, including elevated inflammatory markers and urinary findings consistent with urosepsis. He was treated with appropriate antimicrobial therapy. The infectious process was considered a concurrent illness rather than evidence of a specific GBS trigger.

Neurology reassessment was done due to the progressive weakness, generalized areflexia, respiratory involvement, and the clinical course not being fully explained by the spinal imaging abnormalities. One week after presentation, lumbar puncture was done, demonstrating albuminocytologic dissociation, with CSF protein of 1.46 g/L and one white blood cell/µL (Table 1).

**Table 1:** Selected clinical and laboratory findings during hospitalization. CRP, C-reactive protein; WBC, white blood cell; ALT, alkaline aminotransferase; AST, aspartate aminotransferase; APTT, activated partial thromboplastin time; CSF, cerebrospinal fluid.

Parameter	Patient value	Reference range	Interpretation
Heart rate	110 beats/min	60–100 beats/min	Tachycardia
Temperature	38.9 °C	36.1–37.2 °C	Fever
CRP	252 mg/L	<10 mg/L	Markedly elevated
WBC count	13.5 ×10³/µL	4.0–11.0 ×10³/µL	Leukocytosis
Absolute neutrophil count	10.5 ×10³/µL	1.5–8.0 ×10³/µL	Neutrophilia
Sodium	133 mmol/L	135–145 mmol/L	Mild hyponatremia
Albumin	32 g/L	35–50 g/L	Reduced
ALT	74 U/L	7–56 U/L	Elevated
AST	43 U/L	10–40 U/L	Mildly elevated
APTT	36.2 s	25–35 s	Mildly prolonged
CSF protein	1.46 g/L	0.15–0.45 g/L	Elevated
CSF white blood cell count	1 cell/µL	0–5 cells/µL	Within reference range

NCS demonstrated severe motor and sensory axonal polyneuropathy involving both the upper and lower limbs (Table 2). The study was interpreted as abnormal study showing severe sensorimotor axonal polyneuropathy, which could be seen in diabetic neuropathy. Given the patient's long-standing diabetes, diabetic polyneuropathy was considered an important confounding factor. Nevertheless, in the context of acute progressive weakness, generalized areflexia, respiratory involvement, and albuminocytologic dissociation, the neurology team considered the overall clinical presentation most consistent with a GBS variant within the acute motor axonal neuropathy/acute motor and sensory axonal neuropathy (AMAN/AMSAN) spectrum. 

**Table 2:** Nerve conduction study findings demonstrating severe sensorimotor axonal polyneuropathy. CMAP, compound muscle action potential; EDB, extensor digitorum brevis; TA, tibialis anterior. F wave, late motor response elicited by supramaximal peripheral nerve stimulation.

Nerve	Side	Finding	Result
Median motor	Left	Distal latency	Prolonged (4.8 ms)
Median motor	Left	CMAP amplitude	Reduced (2.1 mV)
Peroneal motor (EDB)	Left	Response	Absent
Peroneal motor (EDB)	Right	Response	Absent
Peroneal motor (TA)	Left	Distal latency	Prolonged (4.5 ms)
Peroneal motor (TA)	Right	Distal latency	4.1 ms
Tibial motor	Left	CMAP amplitude	Markedly reduced (0.1 mV)
Tibial motor	Right	CMAP amplitude	Reduced (0.3 mV)
Ulnar motor	Left	Conduction velocity	Reduced (45 m/s)
Ulnar motor	Right	Conduction velocity	Reduced (43–46 m/s)
Median sensory	Left	Peak latency/amplitude	Prolonged/reduced (5.0 ms; 4.0 µV)
Ulnar sensory	Left	Amplitude	Reduced (6.8 µV)
Ulnar sensory	Right	Response	Absent
Superficial peroneal sensory	Bilateral	Response	Absent
Sural sensory	Bilateral	Response	Absent
F wave – median	Left	Latency	Prolonged (32.95 ms)
F wave – ulnar	Left	Latency	Prolonged (35.00 ms)
F wave – peroneal	Right	Response	Absent
F wave – tibial	Bilateral	Response	Absent

Plasma exchange was administered in six sessions on alternate days, resulting in gradual improvement in limb strength and respiratory function. Following physical and occupational therapy, neurological examination showed 4/5 power in all four limbs with residual right-sided hand-grip weakness. Vibration and joint-position sense remained impaired bilaterally in the lower limbs, with no cerebellar signs. The patient was on room air and discharged with an outpatient neurology follow-up appointment.

## Discussion

The coexistence of significant cervical spinal cord pathology and an acute peripheral neuropathic process reflects the diagnostic challenge. The patient's initial presentation after a fall, urinary incontinence, and MRI evidence of C5-C6 spinal cord compression with T2 hyperintensity raised concern for acute cervical cord pathology [5]. Therefore, cervical abnormalities should not be interpreted simply as an incidental finding or a radiological mimic.

Several subsequent clinical features, however, supported an additional peripheral neurological process. The patient developed progressive weakness in both the lower and upper limbs, generalized areflexia, respiratory compromise, and persistent sensation without a clearly defined sensory level. The development of generalized areflexia favored peripheral neuropathy over cervical myelopathy [6,7]. Neurological reassessment was needed when the weakness progressed, with respiratory involvement, and when the structural abnormalities on MRI did not fully explain the clinical course [8].

CSF showed albuminocytologic dissociation, with elevated protein (1.46 g/L) and only 1 white blood cell/µL, supporting GBS [9]. NCS demonstrated severe sensorimotor axonal polyneuropathy affecting both the upper and lower limbs, although diabetic neuropathy was considered a potential contributor. Given the acute progressive weakness, generalized areflexia, respiratory involvement, and albuminocytologic dissociation, the overall clinical picture was most consistent with a superimposed GBS variant within the AMAN/AMSAN spectrum [10,11].

Diabetes is an important limitation when interpreting the electrodiagnostic findings. Long-standing diabetes may cause distal sensory abnormalities and therefore could contribute to the absent sensory responses observed on NCS. Accordingly, interpreting the electrodiagnostic findings in conjunction with the clinical course and CSF findings rather than as independently diagnostic of GBS is essential [12].

The patient's respiratory deterioration also occurred in the setting of documented infection, including urosepsis and respiratory symptoms. These infectious findings provide an alternative explanation for the fever, tachycardia, elevated inflammatory markers, and respiratory deterioration and should therefore not be presented as diagnostic evidence of GBS. The history of cough and fever before hospitalization represents a possible antecedent infectious exposure; however, a causal relationship between the infection and GBS cannot be established from this case [13].

This case also illustrates the difficulty of distinguishing GBS from cervical myelopathy and traumatic spinal cord injury. Structural abnormalities on spinal MRI are common, particularly in older adults, and their presence does not necessarily establish that they account for the entire neurological syndrome [14]. Conversely, objective spinal cord abnormalities should not automatically be dismissed when peripheral neuropathy is subsequently identified. Clinical assessment of myelopathy requires localization of neurological findings to the spinal cord and correlation with imaging findings. In patients with acute or progressive weakness, serial neurological examinations, including assessment of deep tendon reflexes, tone, pathological reflexes, sensory level, sphincter function, and respiratory status, are essential [15].

An additional consideration is the possibility of concomitant central and peripheral nervous system pathology. In this patient, the cervical MRI demonstrated significant cord compression and T2 signal abnormality, while the clinical course and CSF findings supported a superimposed peripheral neuropathic process. Thus, the most clinically appropriate interpretation is not necessarily that GBS completely mimicked cervical myelopathy, but that GBS occurred in a patient with significant pre-existing cervical spinal pathology and complicated the interpretation of his acute neurological presentation [16].

Three case reports of GBS with concomitant cervical myelopathy or cervical trauma have been published (Table 3) [17-19]. However, the acute presentation of both cervical cord pathology and GBS neuropathy in our case represents a particularly challenging scenario. The history of recent trauma, urinary incontinence, cord compression on imaging, and lumbar stenosis provided plausible explanations for the patient's neurological deficits. The subsequent development of areflexia, limb weakness, respiratory deterioration, albuminocytologic dissociation, and severe motor and sensory deficits supported an additional peripheral cause. The combination of clinically significant cervical myelopathy, lumbar spinal disease, and acute GBS neuropathy created a diagnostic and localization challenge.

**Table 3:** Comparison of published cases of Guillain-Barré syndrome with concomitant cervical myelopathy and the present case. F, female; M, male; AAD, atlantoaxial dislocation; AMAN, acute motor axonal neuropathy; AMSAN, acute motor and sensory axonal neuropathy; CSM, cervical spondylotic myelopathy; CSF, cerebrospinal fluid; GBS, Guillain-Barré syndrome; IVIG, intravenous immunoglobulin; NCS, nerve conduction studies; NR, not reported; OPLL, ossification of the posterior longitudinal ligament; SCI, spinal cord injury.

Author, year	Age/sex	Spinal pathology/trauma	Initial diagnosis	Key GBS features	CSF	NCS/EMG	GBS subtype	Treatment	Outcome
Diwan et al., 2025 [17]	Child/F	AAD, os odontoideum, dens fracture	GBS; later cervical myelopathy	Ascending weakness; bulbar/respiratory involvement; areflexia	Albuminocytologic dissociation	Motor axonal neuropathy	Axonal variant	IVIG; respiratory support; fusion	Full recovery
Miyamoto et al., 2022 [18]	63/M	OPLL; C4 compression after impact	Cervical SCI	Weakness; areflexia; facial involvement; respiratory failure	Albuminocytologic dissociation	Demyelinating NCS; anti-GD1b positive	Demyelinating GBS	IVIG	Full recovery
Neaville et al., 2024 [19]	67/F	CSM; no trauma	CSM	Progressive weakness; areflexia	Supportive	NR	NR	GBS treatment	Initial improvement; later deterioration
Our case	55/M	C5–C6 compression after fall; lumbar stenosis	Cervical spinal pathology	Generalized weakness; areflexia; respiratory involvement	Protein 1.46 g/L; WBC 1/µL	Motor and sensory axonal neuropathy	AMAN/AMSAN	Plasma exchange ×6	Neurological and respiratory improvement

Several lessons can be learned from this report. The acute weakness after trauma in patients with spinal degenerative changes highlights the need for continued reassessments. The progression of neurological symptoms, respiratory deterioration, supportive CSF findings, and electrodiagnostic abnormalities should hint towards the involvement of peripheral neuropathy, while cervical cord abnormalities should be one of the differentials.

## Conclusions

This case illustrates the difficulty of diagnosing acute weakness in a patient with significant cervical spinal disease. Although the cervical MRI findings initially provided a possible explanation for the patient's symptoms, the progressive weakness, generalized areflexia, respiratory involvement, albuminocytologic dissociation, and electrodiagnostic findings raised concern for a GBS variant. The case emphasizes the importance of repeated neurological assessment and integrating clinical findings with imaging, CSF, and electrodiagnostic studies rather than attributing deterioration to pre-existing spinal pathology alone.
